# The ‘SmartNIALMeter’ electrical appliance disaggregation dataset

**DOI:** 10.1016/j.dib.2024.110854

**Published:** 2024-08-19

**Authors:** Manuel Vogel, Martin Friedli, Martin Camenzind, Guido Kniesel, Christoph Klemenjak, Gianni Gugolz, Patrick Huber, Alberto Calatroni, Lukas Kaufmann, Andreas Rumsch, Andrew Paice

**Affiliations:** Lucerne University of Applied Sciences and Arts, Engineering and Architecture, iHomeLab, Horw, 6048, Switzerland

**Keywords:** Non-intrusive load monitoring (NILM), Load disaggregation, NILM datasets, Power signature, Electric load

## Abstract

Electrical disaggregation, also known as non-intrusive load monitoring (NILM) or non-intrusive appliance load monitoring (NIALM), attempts to recognize the energy consumption of single electrical appliances from the aggregated signal. This capability unlocks several applications, such as giving feedback to users regarding their energy consumption patterns or helping distribution system operators (DSOs) to recognize loads which could be shifted to stabilize the electrical grid. The project “SmartNIALMeter” brought together universities, companies and DSOs and involved the collection of a large data corpus comprising 20 buildings with a total of 100 electrical appliances for a period of up to two years at a sampling interval of five seconds. The variability of the loads, including heat pumps and a charging station for electric vehicles, and the presence of single-phase and three-phase devices make this dataset suitable for several investigations. The total consumption was collected through smart meters and each appliance's consumption was measured with a dedicated sensor, providing sub-metering for all loads. The dataset can be used to tackle several open research questions, for example to investigate new NILM algorithms able to learn with a limited amount of sub-metered data.

Specifications Table

**Table:** 

Subject	Energy Engineering and Power Technology
Specific subject area	Measurements of total electrical power consumption from 20 buildings with smart meters and 100 appliances with submeters or on-/off-sensors using a sampling interval of 5 s.
Type of data	Raw data Processed data Filtered HDF5 [1]
Data collection	The aggregated power consumption of each building was measured with the Landis+Gyr (L+G) model E450 3-phase smart meter installed by the utility. Measurements were relayed through the L+G meter Consumer Information Interface (CII) to the Smart-me ‘L+G Module’, which uploaded the data to the Smart-me cloud every 5 s. Single appliance consumption measurements were done with 3-phase meters, single-phase meters or socket-plugs from the vendor Smart-me or with an on/off detection sensor developed by the iHomeLab (iHL). The Smart-me meters copied their measurements every 1 s to the Smart-me cloud and the iHL on/off sensor copied measurements of a 1 s interval to a locally installed Raspberry Pi computer.
Data source location	20 Swiss residential homes in the areas of Zürich, Bern, and Lucerne.
Data accessibility	Repository name: Zenodo Data identification number: 10.5281/zenodo.10875987 Direct URL to data: https://zenodo.org/records/10875988 Instructions for accessing these data: Select link URL to data and download the compressed files “raw.7z” or “preprocessed.7z”. Further instructions can be found on https://github.com/ihomelab/snm-dataset

## 1 Value of the Data

• The “SmartNIALMeter” (SNM) dataset has significant value to the scientific community because of the well-conceived measurement concept. Particularly in the case of load disaggregation, the sampling interval of five seconds and the availability of data of up to two years per household contribute towards training robust NILM models with desirable generalization abilities.
• Although SNM is not the first residential NILM dataset from Switzerland, the data provided by 20 Swiss households allows deep insights into regional characteristics, usage patterns and habits of residents.
• SNM can be used to analyse usage patterns of appliances and trends in electricity consumption, from which conclusions on conserving energy and increasing energy efficiency could be drawn.
• Besides wide-spread appliance types such as fridges and dishwashers, SNM provides energy consumption data of a few EV chargers and heat pumps, which is rarely found in related work.

## 2 Background

In the global push for decarbonization, Switzerland aims to enhance energy efficiency and transition to renewable energy sources (RES) by 2050, focusing on electrifying sectors traditionally dependent on fossil fuels such as transportation and heating. This shift introduces challenges due to large electrical loads and fluctuating RES production, complicating grid management. The SNM project [2] addresses these issues by exploring NILM, a concept pioneered by Hart in 1985 [3,4]. NILM techniques analyse aggregate electrical consumption from a central point, such as a smart meter, to identify individual appliance usage and energy consumption without requiring direct measurement. NILM unlocks two sets of applications: (a) Distribution system operators (DSOs) can infer the presence of specific appliances which might be suitable for peak shaving. For instance, activating a heat pump with a brief delay can go unnoticed, yet this timing adjustment can play a crucial role in lowering peak demand on the electrical grid. (b) Individuals, companies and building administrators can receive feedback about the energy consumption of certain appliances to help reduce the consumption or to diagnose faults. The SNM project contributed by creating a dataset reflecting Swiss energy peculiarities, facilitating the development and validation of NILM algorithms.

## 3 Data Description

### 3.1 Data

For the residential data we chose the Hierarchical Data Format (HDF5), which has been developed for big datasets and fast access [1,5]. We publish two versions of the SNM dataset - a raw version with minimal curation steps and a version with more extensive preprocessing applied. Both versions of the dataset are organized along the same structure: Appliances are saved individually as HDF5 and grouped according to the building they belong to. Measurements from individual phases are denoted by the ending L1, L2 or L3 in the file header (e.g. active power L1). This leads to the following file structure: *<type>/building_<x>/<appliance>.h5*, where:

• *<type>* denotes the type of the dataset, i.e. raw or preprocessed.
• *<x>* is a unique integer assigned to the building.
• *<appliance>* is the name of the measured appliance. The naming follows the NILM metadata convention [6].

### 3.2 Metadata

The provided metadata is identical for both datasets. It follows the NILM metadata convention [6], which is documented in [7]. The metadata is organized in multiple yaml files that can be found in the directory *metadata* in the repository *github.com/ihomelab/snm-dataset*. The metadata is distributed into three different file types with decreasing generality:

1.*dataset.yaml* contains metadata relevant to the whole dataset, e.g. the location, a short description, the publication date and the creators.

2.The file *meter_devices.yaml* describes all sensors used in the measurement process. This includes information about the device model and the manufacturer, a short description, the sampling interval, the type and ratings of the measurements.

3.*building<x>.yaml* models the measurement setup of the respective building. Our measurement setup is described in the form of a tree, where at the root there is the main meter, and the leaves are the sub-meters. This file is organized as follows:

• *elec_meters* contain the list of sensors installed in the building and how they are connected to the others. Every sensor has a number associated with it: The numbers one to three denote the three phases of the site meter, i.e. the smart meter measuring the building's aggregated consumption. These three metering points are marked by the variable *site_meter = true*. Every other sensor is a sub-meter of one of these three sensors/phases. This is specified with the variable *submeter_of*. The model used for metering and the path to the data file is also specified for every sensor. If the time where there is data present of the sub-meter deviates from the timeframe of the smart meter (see item timeframe below), this is also specified.
• *appliances* contain the information about the sub-metered appliances. The NILM metadata convention issues a controlled vocabulary for the naming of the appliances which can be found on this GitHub page [8]. For every appliance the corresponding sensor is mentioned, together with the appliance type.
• *timeframe* gives the period when data are available from the smart meter. The sub-meters inherit this timeframe, if not specified otherwise.

Please note that this setup builds on inheritance: For instance, values that are not provided in *building<x>.yaml* are inherited from *dataset.yaml*, i.e. the geolocation is the same as provided in the file *dataset.yaml* for all buildings.

### 3.3 Heatmaps

**Fig. 1:**
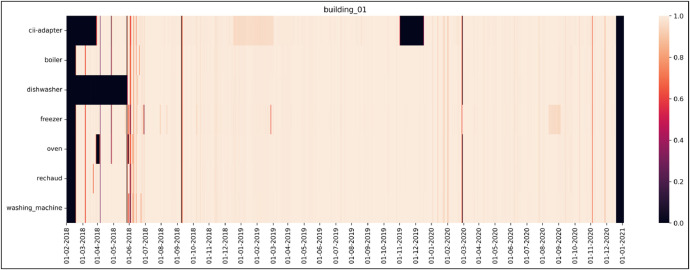
Overview of the data available within the recordings of building_01. The colour coding expresses the percentage of data which are present for a certain time slot. The brighter a region the higher the percentage of the available data. All 20 heatmaps are available in the repository *github.com/ihomelab/snm-dataset* in the *heatmaps* directory.

Heatmaps have been created for each building, showing the percentage availability of data for each device in the corresponding building. Fig. 1 shows an example for building 01.

## 4 Experimental Design, Materials and Methods

**Fig. 2:**
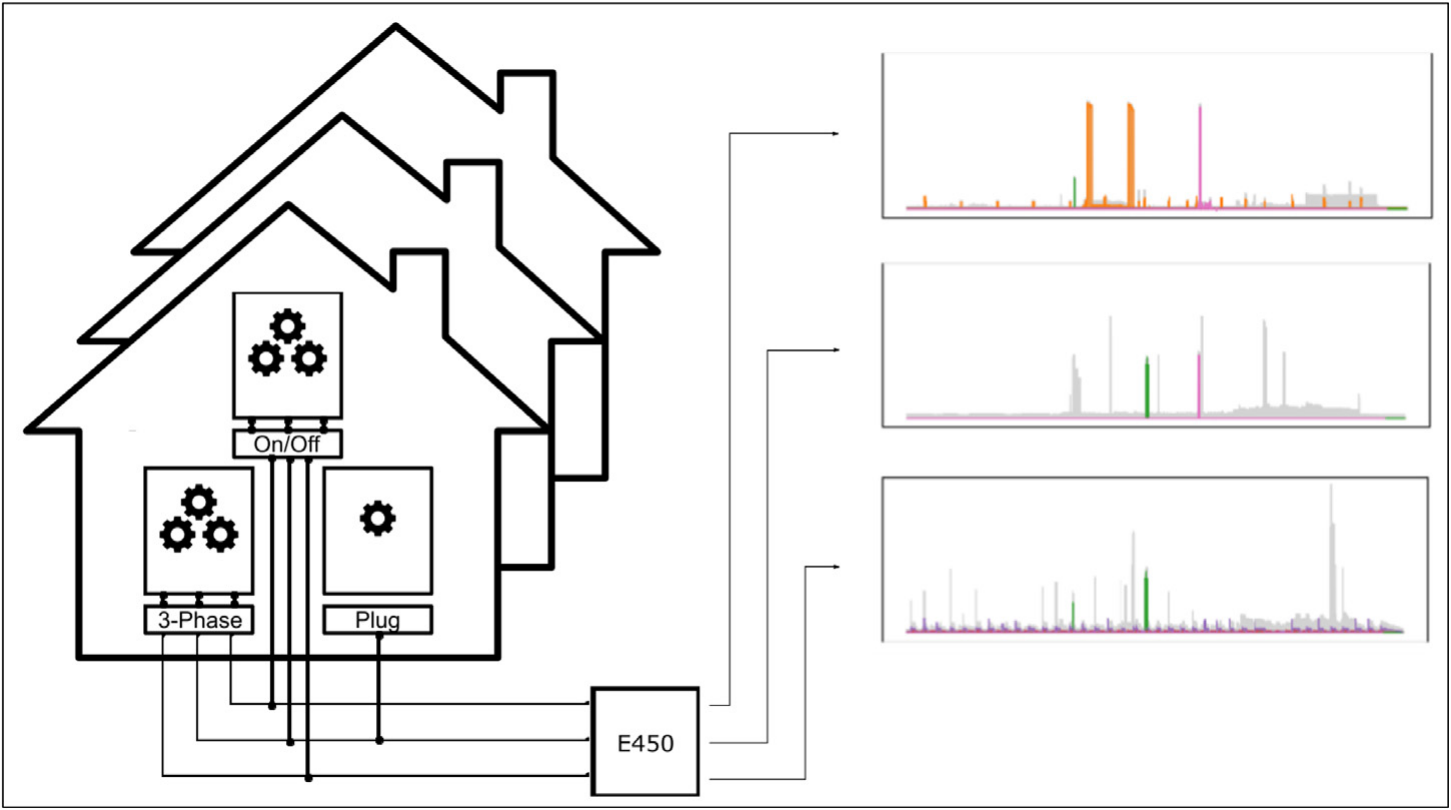
Schematic overview of the study design. The power consumption of various households in Switzerland has been metered with a L+G E450 smart meter. This meter records the power consumption on all three phases individually. Various appliances have been sub-metered to record either their respective power consumption on a single or all three phases or their on/off state.

The data collection has been carried out in two phases. We first deployed the measurement setup to seven buildings (numbers 1 to 7). During this phase we improved the measurement and data acquisition chain. More installations were subsequently rolled out in the pilot phase (buildings 8 to 20). The following sections give a detailed description of the measurement equipment, the data collection processes and the data preprocessing steps. Fig. 2 shows a schematic overview of the study design.

### 4.1 Measurement equipment

**Fig. 3:**
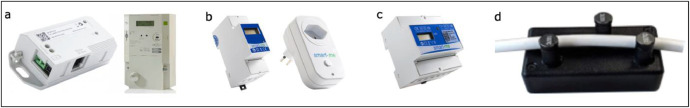
Employed sensors: a) left Smart-me L+G module, right L+G E450 smart meter. b) The two single-phase sensors (left Smart-me single phase, right Smart-me plug) c) Smart-me three phase sensor. d) the developed iHL on/off sensor.

In each building, we measured the overall electrical power consumption and that of specific appliances. The list of pieces of equipment used to collect the dataset, along with detailed specifications and images, is presented in Table 1 and in Fig. 3.

**Table 1:** Overview of the deployed sensors used for data sampling. All Smart-me devices upload data directly to the Smart-me cloud. The on/off sensor relays the data to a Raspberry Pi that uploads it to a Google cloud. Additional information on Smart-me devices and the L+G smart meter can be found at [9,10].

Device name	Details	Measures	Rate
L+G E450 + Smart-me L+G Module	The L+G E450 is the smart meter used to measure the aggregated power, whereas the Smart-me L+G module acts as interface to integrate these readings into the Smart-me cloud. It uploads the root mean square (rms) of the current (Irms), voltage (Vrms) and power factor (cos ϕ) measurement for each phase every five seconds to the Smart-me cloud.	Vrms, Irms, cos ϕ	0.2 Hz
Smart-me plug	A single-phase meter from Smart-me. It is inserted between the application and the socket and measures active power (P) and power factor (cos *ϕ*) every second and uploads the data to the Smart-me cloud.	P, cos *ϕ*	1 Hz
Smart-me single-phase	A single-phase meter from Smart-me that is mounted on a DIN rail. It measures active power (P) and power factor (cos *ϕ*) every second and uploads the data to the Smart-me cloud.	P, cos *ϕ*	1 Hz
Smart-me 3-phase	A three-phase meter from Smart-me that is mounted on a DIN rail. It measures active power (P) and reactive power (Q) every second and uploads the data to the Smart-me cloud.	P, Q	1 Hz
iHL on/off sensor	A low-cost magnetic field sensor developed at the iHomeLab for this project. It measures the magnetic field around the power cable every second and sends it to a Raspberry Pi. This data is used to determine if the application is on or off.	1/0	1 Hz

### 3.2 Aggregate consumption

The aggregate power consumption of each building was measured with the smart meter installed by the utility company. All smart meters were Landis+Gyr model E450. This type of meter provides rms voltage and current values and the phase angle for all three phases every five seconds. Measurements are relayed through the L+G Consumer Interface (CII) to the Smart-me ‘L+G Module’, that feeds the data to the Smart-me cloud. The module is connected to the internet through a Wi-Fi link.

### 4.3 Appliance consumption

Measurements of single appliance consumption (also called sub-metering in the NILM jargon) were performed with different sensors. For single phase appliances, we deployed three types of devices:

• Smart-me single-phase meter. This is a commercially available device which is mounted in the electrical panel, attached to the DIN rail. This device is placed in series with the current flow and measures the active power and phase angle.
• Smart-me plug. This device is very similar to the previous one but is mounted in series to the power cord of the appliance between the plug and the wall socket. It also measures the active power and phase angle.
• iHL on/off sensor. We developed this cheap sensor system to detect whether an appliance is in the ON or OFF state. Generally, due to the proximity of Phase and Neutral in an AC power cord, the magnetic field cancels itself in the far field. However, close to the power cord there exists a residual magnetic field due to the asymmetry of the cable assembly. Our sensor is equipped with three small inductors which measure the residual magnetic field at three different locations close to the surface of the power cord. This magnetic field is proportional to the current flowing in the cable and therefore – assuming a fixed grid voltage – proportional to the apparent power of the appliance. By setting a manually configured threshold we were able to estimate the ON and OFF state of an appliance. The sensor connects via Bluetooth Low Energy (LE) to a nearby Raspberry Pi which takes care of further processing.

We rolled out as many commercial devices as we had budget for. During the pilot phase, some of the single-phase measurements were conducted with the iHL on/off sensor.

For three-phase appliances, such as stoves, ovens, boilers and some washing machines, we used the ‘Smart-me 3-phase meter’, which is mounted in the electrical panel exactly like its single-phase counterpart.

Table 2 shows an overview of the devices and meters used in each building for different appliances.

**Table 2:** Overview on the appliances measured in each building and on the employed meter. Each row corresponds to a building, columns list the available appliances. The naming of the applications follows the NILM metadata convention [6].

buildings	total consumption	washing machine	fridge	freezer	tumble dryer	boiler	stove	dish washer	heat pump	oven	EV charging station	comfort ventilation	coffee machine	dehumidifier	total
1	L+G	3		P		3	3	3		3					6
2	L+G	P	P			3	3	P						P	6
3	L+G	3	P		3	3	3	3		3					7
4	L+G	P	P		P		3*	P		*	3, 3				7
5	L+G	P	P		P		3	P				1	P		7
6	L+G	3	P		P	3	3	P				P			7
7	L+G	3*	P, P	P	*	3	3	3							7
8	L+G	P	P	P	P										4
9	L+G		P	P			O								3
10	L+G	P	P	P	P		O								5
11	L+G	P	P	P	P	O									5
12	L+G	P		P			O	P	O						5
13	L+G		P	P							P				3
14	L+G	P	P	P	P										4
15	L+G	P		P	P			P							4
16	L+G	P	P	P	P				O						5
17	L+G			P	P										2
18	L+G	P	P	P	P				O						5
19	L+G	P	P	P	P			P	L+G						6
20	L+G			P	P										2
total		16	16	15	14	6	10	10	4	2	3	2	1	1	100

Letters in the table have the following meaning: L+G → Landis+Gyr E450 smart meter combined with the Smart-me L+G module; P → Smart-me plug; 1 → Smart-me single phase meter; 3 → Smart-me three phase meter; O → iHL on/off sensor. Buildings 4 and 7 have two appliances of the same type indicated by comma separated letters. The last column and row give the total numbers of the sub-metered appliances. For devices marked with *, the same submeter was used for two appliances in the building, whereby only the first occurrence is counted in the table.

### 4.4 Technical validation

The measurement instruments provided by L+G and Smart-me fulfill the requirements enforced by the Swiss regulations on the measurement of electrical energy and power, which define three precision classes ‘A’, ‘B’ and ‘C’ [11]. The reliability of our measurements can be categorized as follows:

• The L+G E450 smart meters fulfill class ‘B’ precision and have therefore a maximum relative error of 1 %.
• All Smart-me meters fulfill class ‘B’ requirements and have a maximum relative measurement error of 1 %.
• The iHL on/off sensor was thoroughly tested and the threshold on the analog-to-digital converter (ADC) values recorded from the magnetic field measurements was carefully adjusted until the data did not show any mistakes in the on/off signal.
• The time synchronization of the devices attaching timestamps to the samples was obtained by synchronization with network time protocol (NTP) servers. Typical variations in the synchronization are in the order of a few milliseconds and rarely reach a few dozens of milliseconds.
• Despite our best efforts to avoid it, several data are missing. The fraction of available data is depicted in heatmaps for each building (see example for building_01 in Fig. 1).

### 4.5 Data collection

**Fig. 4:**
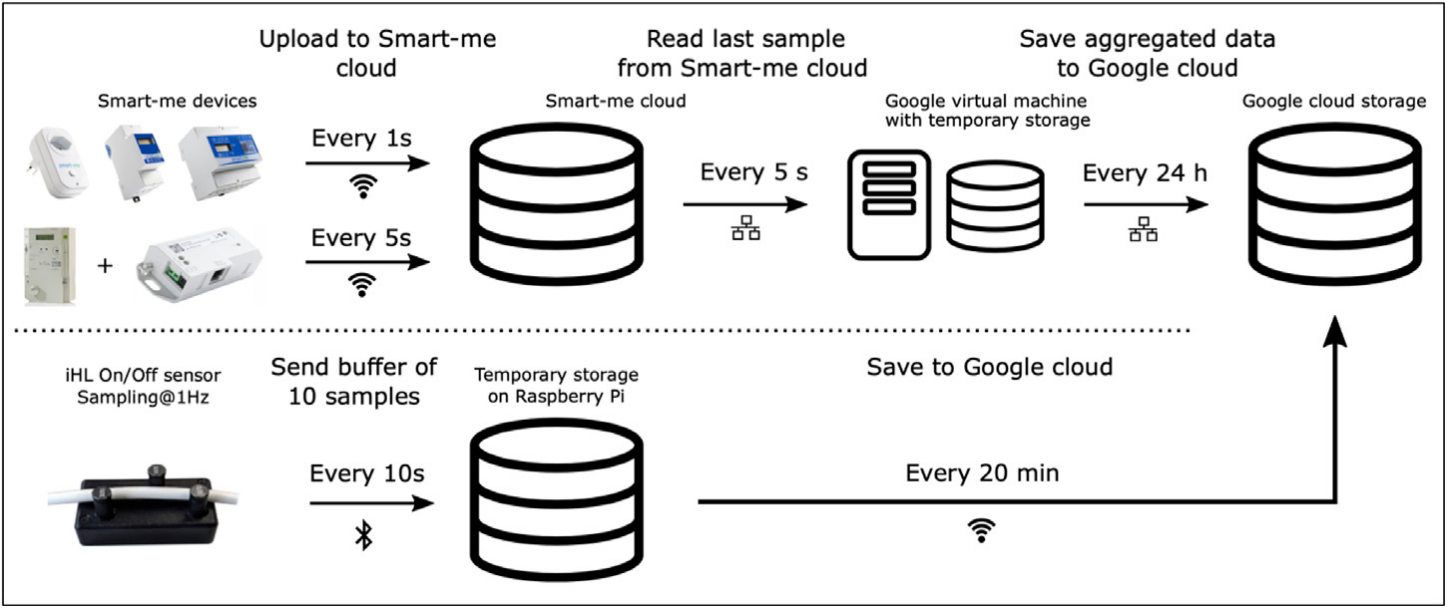
Schematic overview of the data collection process. The upper half depicts the data flow for the Smart-me devices and the lower half for the iHL on/off sensor.

In this section we describe the hardware part of the measurement infrastructure. We now illustrate how the data were gathered. The initial part of the data path is specific to the sensor type, while afterwards all data get centralized to the Google cloud storage. A schematic overview is provided in Fig. 4.

### 4.6 Smart-me devices

The Smart-me L+G module uploads one measurement point every five seconds to the Smart-me cloud. The sensors for sub-metering at the appliance level upload one measurement point per second. In the Smart-me cloud, the data points are stored together with the UTC timestamp measured upon reception of each sample. The Smart-me cloud offers a live view of these data through a web interface. However, historical data are stored only with 15 minutes temporal resolution. To store the higher-rate data, we set up a virtual machine on the Google cloud that requests continuously the newest samples from the Smart-me cloud using their API. Samples from all Smart-me devices are pulled every five seconds irrespective of the original sampling frequencies. On the virtual machine, samples are appended to comma-separated value (CSV) data files. Once per day, the CSV files are compressed and stored in a Google bucket. The file names contain the measurement date.

As it appears clear from the setup, samples obtained from individual sensors are not synchronized: The virtual machine always requests the newest measurement from the Smart-me cloud, which can be up to five seconds in the past in the case of the L+G module and one second in the case of the Smart-me sub-meters.

The described data collection process exhibited occasional connection issues: First, if a sensor loses its wireless connection, no new sample is uploaded to the Smart-me cloud and the newest value on the cloud remains unchanged. Therefore, the virtual machine repeatedly reads the same value(s), resulting in duplicated samples in the CSV file. These values can be easily filtered out since the timestamp itself also remains unchanged. In the raw dataset, these connection issues show up as periods with missing data. Second, whenever the virtual machine loses the connection to the Smart-me cloud, it cannot read any new value, resulting in missing values in the CSV file. To detect these problems, we set up a script on the virtual machine that checks for duplicate and missing values and sends a daily report. From the reports received, we were able to see if there was a need to reboot any of the wireless routers or to reestablish the connection to the Smart-me cloud. Fig. 1 illustrates the data availability using building 01 as an example.

The virtual machine reboots every morning at 02:15 local time (CET). This ensures that all the tasks that read the data from the Smart-me cloud are rebooted and are running properly for the next day. However, since no new data can be read from the Smart-me cloud while rebooting, this leads to a data loss of about 30 seconds. As time stamps of the released dataset are given in UTC time, this reboot happens at 00:15 UTC during the Central European Summer Time (CEST) between the last Sunday of March and the last Sunday of October and at 01:15 UTC during Central European Time (CET).

### 4.7 iHL on/off sensors

The iHL on/off sensors use a different data logging process. Each sensor records the magnetic field strength around the cable every second. Every ten seconds, the buffer containing ten samples x0 . . . x9 is sent over a Bluetooth LE connection to a nearby Raspberry Pi. On the Raspberry Pi, the corresponding timestamps t0 . . .t9 are set so that the newest timestamp t9 is the time measured upon reception, and all previous timestamps are decreased, i.e., ti−1 = ti − 1∀i ∈ [1,9]. To reduce synchronization issues, the Raspberry Pi is synchronized with a NTP server. The time/value pairs are then appended to a local CSV file. A periodic process (cronjob) on the Raspberry Pi synchronizes the locally stored files every 20 minutes with the Google cloud through the rsync Linux command. Data from the iHL on/off sensors are not compressed. To minimize the risk of data loss, a copy of the data is also saved locally on the SD card of the Raspberry Pi.

### 4.8 Privacy preservation

To ensure privacy, all sensors are identified with a random id on the Google cloud. This id can only be mapped to the according metadata such as sensor type and building with the help of a local configuration file. The data storage is organized in a hierarchical manner: Data from every building is collected in a distinct directory, wherein every sensor has its own subdirectory, containing all the daily CSV files.

### 4.9 Processing of measured data

We published two versions of the dataset: The raw version was subjected to basic data curation steps, while the preprocessed version underwent outlier removal and some further feature extraction. Because of the large amount of data, we use dask [12] to efficiently parallelize the data processing. The code used to generate the preprocessed version is available on *github.com/ihomelab/snm-dataset* in the *src* folder.

### 4.10 Raw dataset

We performed the following steps to obtain the published raw dataset:

• In some buildings, the measurement infrastructure was changed during the data collection. In such cases, the sensor id changed and the data before and after the change were saved in two different folders, even if belonging to the same appliance. These cases were dealt with by manually copying the data to the appropriate directory.
• All duplicated, incomplete and not-a-number (NaN) values are deleted from the data records.
• The raw measurements of the iHL on/off sensor, which are acquired through an ADC from the magnetic field, are turned into a binary on/off signal and the original ADC values are discarded. This is done by applying a fixed threshold to the ADC data. The threshold was tuned to be slightly above the noise floor and proved to be effective in the development of the sensor.
• The data are saved to multiple HDF5 files as discussed in the section Data Description.

### 4.11 Preprocessed data

The preprocessed dataset is obtained from the raw data with the following procedure:

• The different Smart-me sensors provide different outputs (see Table 1). We used the following equations to calculate the active power P, reactive power Q and apparent power S for all sensors:
  $$P\;=\;S\;\cdot \;\text{cos}\;\phi \;Q=\sqrt{S^{2}-P^{2}}\;S=P/\;\text{cos}\;\phi \;=\;V_{\text{rms}}\;\cdot \;I_{\text{rms}}$$

where Vrms, Irms and cos ϕ are rms voltage, rms current and power factor respectively.

• We perform outlier detection and remove the corresponding data points. We flag as outliers all measurement values exceeding the sensor rating and negative active power values.
• The data are then saved to HDF5 files, see the section Data Description.

## Limitations

Limitations exist in the sense of data outages due to the temporary failure of individual routers, meters or Smart-me devices during recording. Therefore, heatmaps have been created for each building, showing the percentage availability of data for each device in the corresponding building. All 20 heatmaps are available in the repository *github.com/ihomelab/snm-dataset* in the heatmaps directory.

## Ethics Statement

The authors confirm that they have read and follow the ethical requirements for publication in Data in Brief. They also confirm that the current work does not involve human subjects, animal experiments, or any data collected from social media platforms.

## CRediT authorship contribution statement

**Manuel Vogel:** Data curation, Formal analysis, Software, Validation, Visualization. **Martin Friedli:** Data curation, Validation, Software, Writing – original draft, Writing – review & editing. **Martin Camenzind:** Investigation, Software, Writing – review & editing. **Guido Kniesel:** Project administration, Writing – original draft, Writing – review & editing. **Christoph Klemenjak:** Writing – review & editing. **Gianni Gugolz:** Data curation, Formal analysis, Software, Visualization, Writing – original draft. **Patrick Huber:** Writing – original draft, Writing – review & editing. **Alberto Calatroni:** Writing – review & editing. **Lukas Kaufmann:** Investigation, Project administration. **Andreas Rumsch:** Conceptualization, Funding acquisition, Project administration, Supervision, Writing – review & editing. **Andrew Paice:** Funding acquisition, Supervision, Writing – review & editing.

## Data Availability

The `SmartNIALMeter' Electrical Appliance Disaggregation Dataset (Original data) (https://github.com/ihomelab/snm-dataset).
